# Improving Retention in Care and Promoting Adherence to HIV Treatment: Protocol for a Multisite Randomized Controlled Trial of Mobile Phone Text Messaging

**DOI:** 10.2196/15680

**Published:** 2020-08-27

**Authors:** Elvis Asangbeng Tanue, Dickson Shey Nsagha, Nana Njamen Theophile, Jules Clement Nguedia Assob

**Affiliations:** 1 Department of Public Health and Hygiene Faculty of Health Sciences University of Buea Buea Cameroon; 2 Department of Obstetrics and Gynecology Faculty of Health Sciences University of Buea Buea Cameroon; 3 Department of Medical Laboratory Sciences Faculty of Health Sciences University of Buea Buea Cameroon

**Keywords:** adherence, antiretroviral, HIV, randomized controlled trial, retention in care, text messaging

## Abstract

**Background:**

The World Health Organization has prioritized the use of new technologies to assist in health care delivery in resource-limited settings. Findings suggest that the use of SMS on mobile phones is an advantageous application in health care delivery, especially in communities with an increasing use of this device.

**Objective:**

The main aim of this trial is to assess whether sending weekly motivational text messages (SMS) through mobile phones versus no text messaging will improve retention in care and promote adherence to treatment and health outcomes among patients receiving HIV treatment in Fako Division of Cameroon.

**Methods:**

This is a multisite randomized controlled single-blinded trial. Computer-generated random block sizes shall be used to produce a randomization list. Participants shall be randomly allocated into the intervention and control groups determined by serially numbered sealed opaque envelopes. The 156 participants will either receive the mobile phone text message or usual standard of care. We hypothesize that sending weekly motivational SMS reminders will produce a change in behavior to enhance retention; treatment adherence; and, hence, health outcomes. Participants shall be evaluated and data collected at baseline and then at 2, 4, and 6 months after the launch of the intervention. Text messages shall be sent out, and the delivery will be recorded. Primary outcome measures are retention in care and adherence to treatment. Secondary outcomes are clinical (weight, body mass index), biological (virologic suppression, tuberculosis coinfection), quality of life, treatment discontinuation, and mortality. The analysis shall be by intention-to-treat. Analysis of covariates shall be performed to determine factors influencing outcomes.

**Results:**

Recruitment and random allocation are complete; 160 participants were allocated into 3 groups (52 in the single SMS, 55 in the double SMS, and 53 in the control). Data collection and analysis are ongoing, and statistical results will be available by the end of August 2019.

**Conclusions:**

The interventions will contribute to an improved understanding of which intervention types can be feasible in improving retention in care and promoting adherence to antiretroviral therapy.

**Trial Registration:**

Pan African Clinical Trial Registry in South Africa PACTR201802003035922; https://pactr.samrc.ac.za/TrialDisplay.aspx?TrialID=3035

**International Registered Report Identifier (IRRID):**

DERR1-10.2196/15680

## Introduction

In patients infected with HIV, viral replication can be effectively suppressed with antiretroviral therapy (ART), allowing the body’s immune system to restore and function adequately [1]. Suppressed viral replication in HIV infection has been proven to dramatically reduce mortality and morbidity rates, leading to improved quality of life and improved perceptions on HIV/AIDS from a death sentence to a manageable chronic disease [2]. Yet, the population effect of ART depends on high coverage and sustained adherence to treatment among people living with HIV [3].

The SMS of the mobile phone is an inexpensive and suitable means of communication that can be used in conveying health messages to persons who own or have access to mobile phones. In 2013, 97% of the world’s population were mobile phone subscribers [4]. Moreover, by 2015, 71 per 100 inhabitants of Cameroon were accessing mobile phones [5].

Study protocols on the use of SMS technology to promote adherence to ART have been developed in Kenya [6], India [7], and Cameroon [8]. In 2005, the World Health Organization spelled out the use of new technologies to assist health care delivery in resource-limited settings [9]. Since then, the SMS has shown promising results in improving HIV health care delivery and communication between health personnel and clients, and as an appointment reminder in trials conducted in Kenya [10], Cameroon [11,12], South Africa [13], and elsewhere [14].

The use of text messages in improving adherence to primary care has been found to be more profitable than phone calls [15]. Patient-centered mobile health (mHealth) interventions have had promising outcomes in sickle cell disease management [16]. Systematic reviews have pointed to the success of mobile phone text messaging interventions in improving medication adherence among people living with chronic diseases [17-19]. These findings suggest the use of SMS as a more advantageous application of the mobile phone in health care delivery especially in communities with increasing use of these devices.

Only a single mHealth intervention has been conducted in Cameroon and included only one hospital and 198 participants for a period of 6 months [11]. This lone Cameroonian mHealth intervention together with those conducted elsewhere [10,13,15] has shown promising results on ART adherence and retention in HIV care. Thus, further research involving patients from different treatment centers, and most especially in the phase of government efforts to fight HIV/AIDS, is warranted in Cameroon. This study is timely as it is based on this strategy to improve and promote ART adherence and retention in care in Cameroon. Furthermore, the study will enhance our understanding of the extent that mHealth intervention promotes healthy behaviors and supports psychosocial well-being among patients receiving treatment for chronic diseases. Therefore, this study will contribute to an improved understanding of which text message frequency can be feasible when, why, and for whom.

The goal of this trial is to determine whether sending weekly motivational text messages through mobile phones versus no text messaging will improve retention in care and promote adherence to treatments and health outcomes among patients receiving HIV treatments over a 6-month period. We hypothesize that sending weekly motivational text message reminders will produce a change in behavior to enhance retention; treatment adherence; and, hence, health outcomes.

Participants will be recruited from two hospitals approved to provide free HIV treatment services in Fako Division of the South West Region of Cameroon.

## Methods

The trial was registered with the Pan African Clinical Trial Registry [20] in South Africa on February 1, 2018. The unique identification number for the protocol is PACTR201802003035922.

### Study Design

The design rests on a three-arm, randomized controlled trial with two intervention arms and one control group. The trial shall be single-blinded where the investigators and participants shall not be blinded to the intervention, whereas interviewers and data analysts will be blinded to group allocation. The intervention shall include the use of mobile cellphone SMS in addition to the standard of care provided to the clients. Participants shall be randomly allocated in a 1:1:1 ratio to one of three arms prior to the beginning of the intervention: (1) once weekly mobile phone SMS, (2) twice weekly mobile phone SMS, and (3) a control group that shall not receive mobile cellphone SMS. Clients will be recruited from the government-approved centers of the Regional Hospital Buea and Regional Hospital Limbe providing HIV treatment services.

### Randomization

**Figure 1:**
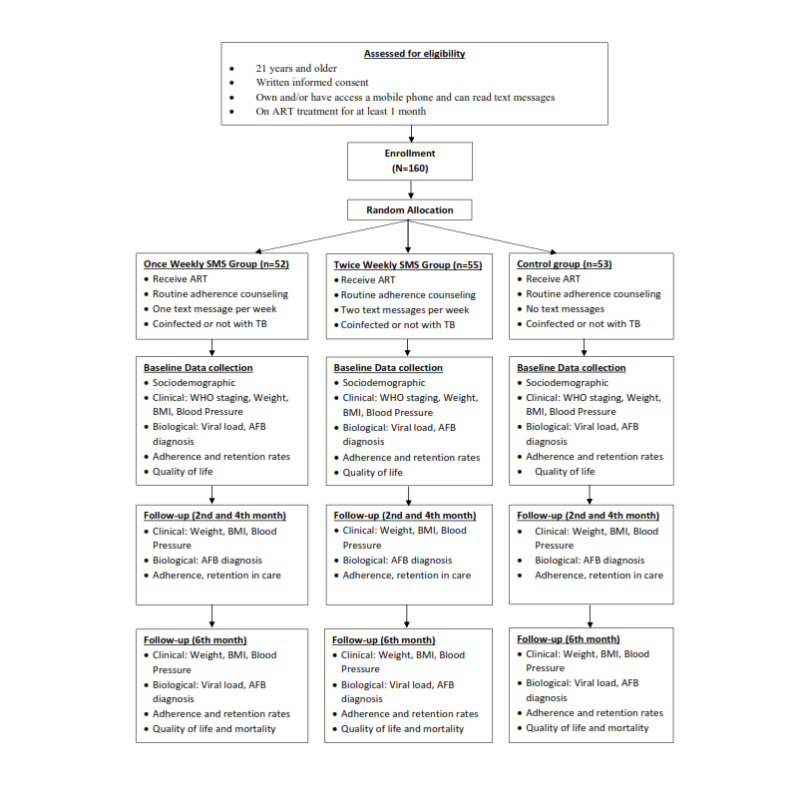
Recruitment and random allocation into the text messaging and control groups. AFB: acid-fast bacilli; ART: antiretroviral therapy; TB: tuberculosis; WHO: World Health Organization.

This is a parallel-group design evaluating the effects of adding once-weekly mobile phone text messages and twice-weekly mobile phone text messages to the usual standard of care (intervention) versus usual standard of care alone (control) among HIV clients receiving ART. Eligible and consenting participants shall be randomized to interventions and control arms using a 1:1:1 allocation ratio by the opaque sealed envelope method. A computer-generated randomization list will be produced using random block sizes of 2, 4, and 6 by the research biostatistician. The allocation codes shall then be put in serially numbered opaque sealed envelopes and administered by the research staff at the various hospitals. Trained interviewers, who shall be blinded to group allocation, will collect data using pretested questionnaires containing sociodemographic data, clinical information, retention, and adherence measures at baseline and at 2, 4, and 6 months. The biostatistician shall also be blinded to group allocation. Blood samples will be collected and analyzed for viral load during the trial once a participant has been on ART for at least 6 months. The time schedule of enrollment, interventions, and assessments are summarized in Figure 1.

### Trial Setting

Cameroon is a sub-Saharan central African country made of ten regions and a population of over 24 million inhabitants [21]. The study will be implemented in Fako Division of the South West Region of Cameroon. The adult prevalence of HIV in the country was 3.4% in 2018 [22]. The adult prevalence of HIV in the South West Region was 3.6% in 2018 [22]. Most inhabitants practice agriculture as the main economic activity. The region has two seasons: the dry season from October to March and the wet season from April to September. Almost all ethnic groups in Cameroon are represented in the region, attracted by the fertile volcanic soil and the Cameroon Development Corporation, a giant agricultural corporation that seconds the state of Cameroon in employment. The two hospitals selected for the trial have government-approved centers offering free treatment and care services for HIV to the population of the region. Participants will be residents in their various communities and support groups while visiting these treatment hospitals for routine care during the entire study period. As per the Cameroonian Ministry of Public Health national guidelines, newly diagnosed clients are initiated on free nonnucleotide reverse transcriptase inhibitors–based ART irrespective of their viral load count or CD_4_^+^ T cell count [11]. Recently, HIV clients initiating ART were systematically placed on isoniazid prophylaxis for the first 6 months of their ART. These are the largest treatment centers in the region and offer enormous potential for recruitment.

### Participants: Inclusion and Exclusion Criteria

The study population shall include HIV clients either coinfected with or without tuberculosis who come to the hospital for a routine health evaluation. Participants shall be consecutively recruited and randomly allocated to the intervention and control groups.

We shall include clients who are 21 years or older, have access to or own a mobile phone and can read text messages, and who have been on ART for at least a month and planned to live in Cameroon during the period of the study. We shall place more emphasis on clients who have started ART for at least 1 month to measure the differential effects for clients initiating ART versus those who have been under treatment for longer durations. Informed consent is a prerequisite for participating in the study and shall be provided orally and in writing.

We shall exclude clients who have been on ART for less than a month and who are younger than 21 years. Clients who have used ART for at least 1 month are chosen to enable us to be able to calculate a baseline figure for treatment adherence.

### Intervention

The HIV clients will be receiving ART for free from the hospital staff at the HIV treatment centers. The HIV clients who shall be coinfected with tuberculosis shall also receive directly observed treatment short-course for free. This is the usual standard of care for people living with HIV/AIDS and tuberculosis in Cameroon [23]. Patients with HIV on any of these treatments shall be randomized to receive mobile phone text message reminders. The trial shall comprise 2 intervention groups and 1 control group to investigate the impact of text message reminders on retention in care and adherence to treatment and health outcomes.

A short text message will be sent to the participants in the intervention groups in English or French depending on the preferred language of each client. The content of these text messages has been developed and pretested in focus group discussions involving people living with HIV/AIDS, their caregivers, and health care workers. The construct of the health belief model was used to develop these text messages that are sociocultural acceptable and targeted at improving retention in care and promoting adherence to treatment [24]. The messages are motivating and shall act both as reminders and a cue to action. Participants unanimously agreed that the messages should not contain the name of the disease. It was also concluded that the word “food” should be used in place of medication or drug when sending the messages (Textbox 1). The message shall also contain a phone number that the participants can call back if they need directives on their treatment and care. The content shall be varied so as to retain participants’ attention and interest throughout the period of the study and to explore the various aspects of behavior change. There shall be a list of phone numbers that the messages targeted at improving retention in care shall be sent midway before the next clinic visit schedule and then 2 days before each clinic visit appointment. The messages targeted at promoting adherence to treatment will be sent twice every week. The “delivery report” function of the mobile phone shall be used to verify and to record whether the messages have been delivered to the clients. Clients will be contacted through the phone numbers of their contact persons in the event of undelivered messages. The issue of missing phone numbers shall be resolved by replacing clients’ existing contracts with their recent phone numbers. One message will be sent per week in the evening between 6 PM and 7 PM of a day chosen by the client in the once-weekly SMS intervention arm. In addition, two messages shall be sent per week in the evening between 6 PM and 7 PM of 2 days chosen by the client in the twice-weekly SMS intervention arm. The mobile phone text messaging shall be provided as add-ons to the usual standard of care, which includes rare ART counseling and occasional home visits.

**Examples of the text messages that will be sent in the intervention group.**

**Sample SMS to improve retention in care**

- You are so loved. Handle your health with care, visit us on xx-xx-xxxx.
- You are very important. Do not forget your visit on xx-xx-xxxx.
- Are you busy? This is why I remind you to come for your visit on xx-xx-xxxx.
- Your health is important. Come for your appointment on xx-xx-xxxx.

**Sample SMS to promote adherence to treatment**

- Your health is important. Take your food.
- You are very important. Do not play with your health. Take your food.
- You are so loved. Handle your health with great care. Take your food.
- Are you very busy? This is why I remind you to regularly take your food.

### Control

The routine practice in Cameroon is that, at ART initiation, the responsibilities of the treatment centers usually explain the side effects of medications and problems associated with poor adherence to the clients prior to dispensing drugs. All participants including those in the control arm have received this educational message during their routine hospital visits. Participants randomly allocated to the control group shall not receive mobile phone SMS. However, they shall receive ART, be screened for tuberculosis coinfection, and be interviewed alongside the other participants.

### Study Objectives

#### Primary Objective

The main aim of this trial is to investigate the impact of adding mobile phone SMS to the usual standard of care versus usual standard of care alone in improving retention in care and promoting adherence to treatment among HIV clients on treatment at 2, 4, and 6 months. Retention in care shall be defined as the proportion of clients who had started on ART, enrolled in the study, and attended clinic visits at the second, fourth, or sixth months. There is no gold standard in the measurement of adherence because the majority of the tools currently used cannot meet all the features of an ideal tool [25]. However, a multi-method tool is recommended and can include self-report and different combinations of other tools including pill count, the pill identification test (PIT) and visual analog scale (VAS), electronic methods, and drug levels. Therefore, a composite adherence measure shall be used in this study to reduce the errors associated with using a single adherence measure [25]. The adherence score shall be built on four adherence measures including a self-report questionnaire with 4 items, supplement of pill pick up from pharmacy refill records, a PIT consisting of 5 items, and a 30-day VAS. Adherence shall first be estimated by each measure and classified into three categories as high, moderate, and low before finally combining the results of the four measures into the composite adherence score.

#### Secondary Objectives

The secondary objectives include comparing health outcomes such as weight, BMI, opportunistic infections such as tuberculosis, and quality of life. These comparisons will be performed between the groups at baseline and at 2, 4, and 6 months. In addition, the viral load will be determined for participants who have been receiving ART for at least 6 months to ascertain virologic suppression.

### Outcome Measures

#### Primary outcomes

The primary outcomes will be retention in care and adherence to treatments, measured using self-reports (supplemented by PIT, pharmacy refill data, and VAS; Table 1).

**Table 1:** Overview of outcome measures.

Outcome measures			Scale	Type	Measure	Analysis method
**Primary**						
	Retention at 2, 4, and 6 months		Nominal	Binary	Number retained in care	Risk ratio
	**Adherence at baseline and at 2, 4, and 6 months**					
		Self-report	Ordinal	Binary	% adherence in last month >95%	Risk ratio
		Pill identification test	Ordinal	Binary	% of pills identified >95%	Risk ratio
		PRD^a^	Ordinal	Binary	% of complete refills >95%	Risk ratio
		VAS^b^	Ordinal	Binary	VAS percentage >95%	Risk ratio
**Secondary**						
	Weight		Ratio	Continuous	Change in weight	*t* test
	BMI		Ratio	Continuous	Change in BMI	*t* test
	Viral load		Ratio	Continuous	Change in viral load	*t* test
	OIs^c^ (AFB^d^ diagnosis)		Nominal	Binary	Occurrence of new OI (AFB positive smear)	Chi-square test
	Mortality		Nominal	Binary	All deaths	Chi-square test
	Satisfaction with care		Ordinal	Categorical	Change in satisfaction scores	*t* test
	QoL^e^		Ordinal	Categorical	Change in QoL scores	*t* test
**Overall**						
			Nominal	Binary	Number retained in each group	Risk ratio
			Ordinal	Binary	Change in composite adherence measure	Risk ratio

^a^PRD: pharmacy refill data.

^b^VAS: visual analog scale.

^c^OI: opportunistic infection.

^d^AFB: acid-fast bacilli.

^e^QoL: quality of life.

#### Secondary Outcomes

The secondary endpoints shall comprise clinical (weight and BMI), biological (viral load and AFB diagnosis), quality of life (measured with the self-functioning–12 quality of life assessment form), and all-cause mortality. Treatment discontinuation, reasons for discontinuation, and risk factors of treatment discontinuation shall also be determined. The trial endpoints are summarized in Table 1.

### Duration

The trial shall run for 6 months. Outcome assessments will be conducted at baseline, 2 months, 4 months, and 6 months.

### Sample Size Determination

The sample size determination is based on the test of the null hypothesis that the rates of adherence to ART in the intervention and control groups are equal. The primary measure of effect is the rate of adherence to ART as measured by using the VAS over 6 months. Given the information from previous studies [10,11], it is estimated that approximately 60% of participants would maintain adherence ≥95% without intervention and that the interventions would help 82% of the participants maintain ≥95% adherence. A two-sided test is assumed where an effect in either direction will be interpreted at a significance level of 5%. The study will have a power of 80% to yield a statistically significant result using a chi-square test (assuming an intention-to-treat principle for the analysis) of the relative risk at an alpha level of 5% to detect a 25% difference (60% vs 85%).

The formula for calculating the sample size for randomized controlled trials by Chan [26] is used.


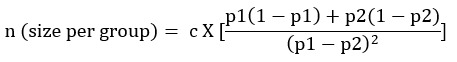


where c=7.9 for 80% power and p1 and p2 are the proportion estimates (60% for the control group and 85% for the intervention group).


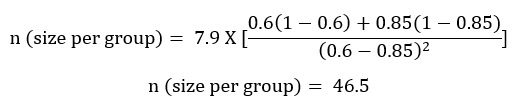


It is assumed that 10% of the participants shall drop out of the study due to loss to follow-up and mortality. A total of 52 participants shall be randomized to each arm to allow for the 10% drop out. Therefore, the required sample size will be 156 (52 x 3 = 156) participants.

### Analysis Plan

The CONSORT (Consolidated Standards of Reporting Trials) guidelines will be used in reporting the results. The biostatistician shall be blinded to the group allocation. The process of client selection and flow throughout the study will be summarized using a flow diagram. The analysis of client demographics and outcome variables shall be summarized using descriptive summary measures, expressed as mean (standard deviation) or median (range) for continuous variables and number (percentages) for categorical variables. All outcomes shall be analyzed using an intention-to-treat principle where data from participants shall be analyzed according to the group to which they were randomized even if they do not receive the allocated intervention. Missing data shall be handled using the multiple-imputation method. The student *t* test and analysis of variance for comparing group means will be used. All statistical tests shall be performed using two-sided tests at the 5% level of significance. The Bonferroni method shall be used to adjust the level of significance for testing of secondary outcomes to keep the overall level at an alpha of 5%. For all group comparisons, the results shall be expressed as an effect (or risk ratio for binary outcomes), corresponding two-sided 95% confidence intervals, and associated *P* values. *P* values shall be reported to three decimal places with values less than .001 reported as <.001. Adjusted analyses using baseline variables shall be performed using regression techniques to determine the continuing influence of key baseline characteristics on the outcomes. The Kaplan-Meier survival analysis will be used for timed variables like mortality. All analyses will be performed using SPSS version 25.0 (IBM Corp) for Windows. Adherence will be measured both as a continuous outcome (change in adherence) and as a binary outcome (ie, adherent, 95% of pills taken, or nonadherent, <95% of pills taken) following the composite adherence score method. Adherence data shall be handled in a number of ways, reported as the number of doses respected, and shall be combined into a composite score. The effects of the intervention shall be reported on all the measures of adherence used and will be compared for discrepancies.

### Patient and Public Involvement

This study was conceived and designed to address gaps in the care and support available to people living with HIV/AIDS who are receiving ART. The intervention was designed with the active involvement of patients with HIV, their caregivers, and health workers through focus group discussions. Our randomized controlled trial offers participants the opportunity to provide feedback regarding the burden of the intervention through focus group discussions involving a cross-section of participants.

### Ethics and Dissemination

Ethical clearance for this trial was obtained from the Institutional Review Board of the Faculty of Health Sciences of the University of Buea in Cameroon (Reference number: 2018/147/UB/SG/IRB/FHS). Administrative authorization was obtained from the Regional Delegation of the Ministry of Public Health for the South West Region and the District Health Services. The purpose of the study and the role of the participants will be well explained in the consent form to the participants and participation shall only take place after the participant has read and signed the informed consent forms voluntarily. The informed consent shall include signed permission to consult the client’s medical records over the duration of the study.

Dissemination efforts shall target individuals and institutions that will have the most impact on local, national, and international HIV and AIDS policies. Therefore, dissemination plans shall include the presentation of research findings in seminars, conferences, and scientific publications in peer-reviewed journals. A summary of the findings will be made available to participants.

## Results

Recruitment of participants in the trial took place in May and June 2018. Recruited participates were randomly allocated to the intervention and control arms at the start of the intervention in July 2018.

A total of 160 participants have been recruited and randomly allocated into the intervention and control groups. Participants were allocated into 3 groups (52 in the once weekly SMS, 55 in the twice weekly SMS, and 53 in the control). Data collection and analysis are ongoing, and statistical results will be available by the end of August 2019.

## Discussion

Since the launch of the fast track approach to end the AIDS epidemic by 2030, Cameroon is far from reaching the 90-90-90 treatment target in 2020, whereby 90% of people living with HIV know their HIV status, 90% of people who know their HIV-positive status are accessing treatment, and 90% of people on treatment have suppressed viral loads [27]. Only 58% of people living with HIV know their HIV status, 37% of those living with HIV are on treatment, and 19% of these patients are virally suppressed 2 years from the 2020 treatment target in Cameroon [27].

New strategies to end the AIDS epidemic as a public health threat by 2030 are being instituted throughout most national HIV control programs [28]. Recent advances in the fast-track approach to HIV and AIDS in Cameroon has been the recommendation of offering to every client presenting to a clinic for any medical consultation to pass the HIV screening test and to allow as many people to know their HIV status [29]. In addition, the government of Cameroon through the Ministry of Public Health has instituted new directions by placing the systematic treatment of HIV under the strategy called test and treat (screening and treatment) [29]. This new strategy requires that any person screened and confirmed positive for HIV is directly placed on antiretroviral treatment. Proper retention in care and a high level of adherence are needed to sustain lower viral load counts and reduce chances of drug resistance that might result from treatment defaulting. More people will be accessing ART services as a result of enhanced HIV testing. There is a high need to devise strategies that would lead to improved retention in care and sustained adherence to treatment.

In this light, the benefits of mobile phone SMS in improving and promoting health outcomes are warranted, particularly in this era of increased uptake and use of mobile phone devices. However, there is a paucity of economic data to support the use of mHealth behavioral interventions in low- and low-middle–income countries [30].

Improving retention in care and promoting adherence to treatments can play a key role in reducing the morbidity and mortality associated with HIV and tuberculosis diseases. The occurrence of drug-resistant strains and the waste of medication in health systems can be adequately managed. Findings generated from this trial may be generalizable to other chronic illnesses requiring lifelong treatments.

A major ethical concern is the harm that might be caused to participants due to the accidental disclosure of their disease status. This possibility shall be properly explained to the participant, even though our text messages shall neither disclose status nor make mention of medication but shall rather act as a reminder of health. Loss of privacy and confidentiality are not foreseeable problems in the study. The intervention shall be an addition to an already existing system in Cameroon where mobile operators deliver messages to their clients for business purposes. The text messages shall be delivered to the participants’ mobile phones by the research team using phones with prepaid airtime.

The content of the SMS was developed following the construct of the health belief model of behavior change. Furthermore, the intervention shall focus on the health belief model. Collected data shall be used to test the efficacy of the SMS reminder as a cue to action. This study is timely, as it is based on this strategy to improve and promote ART and tuberculosis treatment adherence and retention in care in Cameroon. Furthermore, the study will enhance our understanding of the extent that mHealth intervention promotes healthy behaviors and support psychosocial well-being among patients receiving treatment for chronic diseases. The study shall also investigate the effect of tuberculosis coinfection on the health outcomes of HIV clients. Therefore, the study will contribute to an improved understanding of which type of text message frequency can be feasible when, why, and for whom.

## Abbreviations

ART: antiretroviral therapy
CONSORT: Consolidated Standards of Reporting Trials
mHealth: mobile health
PIT: pill identification test
PRD: pharmacy refill data
VAS: visual analog scale
